# Engineered Nanoclusters to Selectively Reduce Mesenchymal and Epithelial Melanoma Cell Viability

**DOI:** 10.3390/cancers17121903

**Published:** 2025-06-07

**Authors:** Olga M. Rodríguez Martínez, Amy Wu-Wu, Valeria S. Arroyo Suárez, Karina Ruiz Rivera, Krystal A. Quirindongo Ortíz, Kiara Y. González Pérez, Miguel E. Castro Rosario

**Affiliations:** 1 Bioengineering Program, College of Engineering, University of Puerto Rico at Mayagüez, Mayagüez, PR 00682, USA; olga.rodriguez9@upr.edu; 2 Department of Biology, College of Arts and Sciences, University of Puerto Rico, Mayagüez, PR 00682, USA; amy.wu@upr.edu (A.W.-W.); valeria.arroyo2@upr.edu (V.S.A.S.); karina.ruiz3@upr.edu (K.R.R.); 3 Department of Chemical Engineering, College of Engineering, University of Puerto Rico, Mayagüez, PR 00682, USA; krystal.quirindongo@upr.edu; 4 Department of Biology, College of Arts and Sciences, Lewis & Clark College, Portland, OR 97219, USA; gonzalezkiara@lclark.edu; 5 Department of Chemistry, College of Arts and Sciences, University of Puerto Rico at Mayagüez, Mayagüez, PR 00682, USA

**Keywords:** melanoma, vinculin, actin, focal adhesion, calcium sulfide, extracellular pH, caspases, intrinsic apoptosis

## Abstract

Melanoma is the most common type of skin cancer. Melanomas are well known for their ability to metastasize to other organs, including the lungs, liver, brain, and bones. The ability of melanoma cells to switch among different phenotypes is a key mechanism that underscores their metastatic potential. The objective of this work is to report here on the effect of calcium sulfide (CaS) dispersions in melanoma cells. Melanomas with the epithelial- and mesenchymal-like phenotypes were observed during cell culture preparation. The dose-dependent viability was explored up to slightly less than 3% per volume of cell culture. The dispersion reduced the relative percentage of melanomas with the epithelial- and mesenchymal-like phenotypes to (57 ± 5) and (55 ± 5)%, respectively, at 24 h post treatment. In contrast, the viability of normal fibroblasts treated with the dispersion or melanoma cells treated with the reactants used to prepare the dispersion remained nearly constant, with a value range of (100.0 ± 0.2)% for the control and (97 ± 4)% and (93 ± 2)% for doses as high as 2 and 3% per volume of cell culture, respectively. Fluorescence imaging measurements were consistent with the release of cytochrome c from the mitochondria and its translocation to the cell nuclei. The average expression of caspases 3 and 9 was found to be 3 and 1.4 times higher than in the corresponding melanoma control, respectively, which was consistent with intrinsic apoptosis. The response of vinculin expression was slightly different in both cell phenotypes. Vinculin was found to delocalize in the cytoplasm of treated mesenchymal melanoma cells, with a slightly higher concentration at the end of the actin fibers. A statistically significant increase (*p* < 0.0001) in the number of focal adhesion points (FAP) at the edge of the cell membrane–external cellular matrix (ECM) interphase was observed in post-treated melanoma that exhibited the epithelial-like phenotype. The changes in vinculin expression and FAP and the reduced viability of the melanomas were consistent with regulation of proteins associated with programmed cell death. It is thus proposed that the sulfides produced from the reactions of the nanoclusters in the acidic environment facilitate the regulation of proteins required to initiate apoptosis, although other processes may also be involved. We conclude that CaS may be an adequate chemical to selectively reduce melanoma viability with little effect on benign fibroblasts.

## Simple Summary

The ability of melanoma cells to switch among different phenotypes facilitates metastasis and evasion of treatments. We use CaS nanoclusters to selectively reduce the viability of epithelial- and mesenchymal-like melanoma phenotypes with no significant effect on benign fibroblast viability. The nanoclusters reduce melanoma viability in melanomas with either phenotypes by intrinsic apoptosis as supported by fluoresccence imagng measurements of translocated cytochrome-c and the increase in caspase 3 and 9 average expression. Interestinly, vincluin was found to delocalize in the cytoplasm of treated melanomas with the mesenchymal-like phenotype. In melanomas with epithelial-like phenotypes, the number of focal adhession points at the cell membrane-extracellular matrix interphase is found to increase compared to the control. The results encourage future preclinical work with animal models.

## 1. Introduction

About 200,000 cases of melanoma have been diagnosed in the USA according to the American Cancer Society. The early state of melanoma is a localized cancer, with a 5-year survival rate of 94%. However, a late diagnosis or intervention may be fatal: melanoma is the most metastatic type of cancer. Indeed, only 32% of patients survive metastasis. Melanoma has the capacity to use the host microenvironment to promote cellular proliferation and tumor growth, switching between different phenotypes. In nature, melanoma is highly angiogenic and mesenchymal [1]. The mesenchymal phenotype has well-defined actin fibers, including dynamic actin-rich protrusions such as filopodia and lamellipodia at their leading edges. These protrusions are involved in cell migration and are powered by the dynamic polymerization and depolymerization of actin filaments. Those in the epithelial state, on the other hand, are characterized by well-organized cortical actin network located just beneath the plasma membrane. This network supports the cell’s shape and maintains a tight, sheet-like structure.

Melanoma cells with epithelial phenotypes play central roles in the formation of localized tumors due to their proliferative characteristics [2]. Melanoma cells with mesenchymal phenotypes are more difficult to treat with chemotherapeutic agents because of higher mobility and metastatic characteristics [2]. The dynamic actin cytoskeleton facilitates this behavior, allowing cells to crawl along surfaces and navigate through tissues, forming focal adhesion points. This diversity may result in a complicated design of chemotherapeutic agents to reduce melanoma cell proliferation and/or metastasis. During melanoma progression, cells may undergo an epithelial-to-mesenchymal transition (EMT), where they lose epithelial characteristics and gain mesenchymal properties. This transition involves significant changes in actin organization, including loss of tight junctions and enhanced stress fiber formation. EMT is a complex process that involves transcription factors and genes [3]. These transitions are bidirectional and play a central role in facilitating melanoma cell migration to nearby and distant tissues and organs [4]. EMT is associated with melanoma metastasis [5].

Cell adhesion and migration depend on intracellular processes and their interactions with the extracellular matrix through important groups of proteins that constitute the focal adhesion points (FAP) [6]. These FAP are crucial for cell migration and are involved in signaling pathways that regulate cell movement and response to the extracellular environment. FAP complexes are formed from the interactions among various proteins and kinases, including vinculin, actin, talin, paxillin, integrin, focal adhesion kinase (FAK), proto-oncogene tyrosine-protein kinase (SRC), and PI3K, among others [4]. In normal cells, regulation of the focal adhesion point formation is largely due to activation and de-activation of several of these proteins [7]. This regulation mechanism plays a significant role in controlling cell death and proliferation [8]. The regulation of proper FAK as well as other kinases requires adequate phosphorylation and de-phosphorylation of tyrosine residues by cysteine residues. Cysteine is usually present in the non-protonated state with a negatively charged sulfur end (C_3_H_6_NO_2_S^−^). Thus, it is plausible that chemical systems that can provide sulfides can enhance regulation of the dephosphorylation of kinases, similar to the sulfide end in cysteine residues that can trickle down, with a reduction in malignant cell viability [9].

CaS (calcium–sulfur) is used as a remedy in alternative medicine to treat colds, coughs, sore throats, croup, abscesses, earaches, inflamed cuts and wounds, asthma, arthritis, emphysema, herpes, constipation, conjunctivitis, *Candida albicans* infections, syphilis, sinusitis, and skin infections [10]. Compared to many popular nanomaterials, including noble metals and magnetic nanoparticles, calcium- and sulfur-based nanostructures are promising biocompatible inorganic materials. Calcium is an abundant mineral in the human body. It plays major roles in both in macroscopic biological functions such as bones remodeling and biological functions at the molecular level such as intracellular and extracellular communication, protein structures, and mitochondrial metabolism. Sulfides, on the other hand, are present in the human body mostly in organic compounds like proteins and genetic material, and they are now postulated as a neuromodulators/transmitter with important physiological properties [11].

The pH of normal and tumor cells depends on the tissue from which the sample is taken. The extracellular pH (pHe) of benign cells is slightly basic and ranges from 7.35 to about 7.8, while the pH_e_ values of cancer cells are more acidic, with values as low as 6.2 [12]. This difference in pH offers a unique opportunity to design and develop technologies that can selectively act on malignant tumors with little effect on benign tissue. In this regard, CaS may represent a unique opportunity to engineer technologies to selectively limit cancer cell viability. In proton-deficient environments, like the extracellular matrix of benign cells, the dissociation of CaS into calcium and sulfide ions is a non-spontaneous process with a standard Gibbs free energy of reaction (ΔG°_rxn_) of +9.2 kJ/mol. In the proton-rich extracellular environment of cancer cells, on the other hand, the reaction of CaS with protons to form calcium ions and protonated sulfides is highly exergonic, with ΔG°_rxn_ that ranges from −109.7 to −103.58, with the exact value depending on the protonated form of sulfide formed. Thus, the formation of Ca^2+^ and H_2_S is thermodynamically favored in the acidic pH found in the extracellular fluid of cancer cells but is limited in the basic extracellular environment of benign cells. Indeed, the experimental measurements reported earlier by our group are consistent with the pH-sensitive release of H_2_S to the gas phase. In that work, the amount of H_2_S detected from CaS was found to decrease with pH and was under detection limits at the more basic pH values [13]. We hypothesized that the difference in pH results in the selectivity of the CaS nanostructures to limit the viability of breast [14,15,16] and lung [17,18] cancer cells, with little effect on the corresponding benign cells. The quantitative results on the effect of CaS on breast and lung cancer cell viabilities are consistent with this hypothesis. We thus extended our earlier work on breast carcinoma and non-small-cell lung adenocarcinoma to skin melanoma with the hope of adding to the body evidence on the selective reduction in malignant cell viability by CaS.

## 2. Methodology and Approach

### 2.1. Cell Culture

Human Fibroblast cells CCD1090Sk (ATCC^®^ CRL2106™; Manassas, VA, USA) and Melanoma cell line Hs 895.T (ATCC^®^ CRL7637™) were passaged and maintained in complete cell media. The complete medium had a final composition of 89% Minimum Essential Medium (EMEM; ATCC) and Dulbecco’s Modification of Eagle’s Medium (DMEM; ATCC), respectively, with 10% Fetal Bovine Serum (FBS; ATCC) and 1% of Antibiotic Antimycotic Solution (Penicillin/Streptomycin/Amphotericin B solution), as supplied by ATCC; PCS-999-002. The experiments described below were performed with cells at 60–80% confluency.

### 2.2. CaS Dispersion Preparation

CaS dispersions were prepared as described in previous [19,20]. Briefly, a trace amount of laboratory grade calcium acetate (Ca (CH_3_CO_2_)_2_), purchased from Fisher Scientific, was dissolved with 5 mL of dimethyl sulfoxide (DMSO), and the resulting dispersion was placed in a commercial microwave oven and warmed in intervals of 5 s until a total time of 30 s was accumulated. The resulting dispersion was found to be slightly yellow. The dispersions were characterized with scanning tunneling electron microscopy (STM), light scattering, and UV–visible absorption spectroscopy. STM measurements of dry deposits of the prepared material revealed structures with an average size of (3.3 ± 0.7) nm, while the light scattering measurements were consistent with particles smaller than (1.1 ± 0.2) nm: the larger particle size from the STM measurements likely results from coalescence of the dry deposits. Theoretical calculations focused on particle size volume were consistent with clusters containing a handful of CaS units were of the order of 1 nm [18]. Features in the UV–visible absorptions spectra were also consistent with the optical absorption characteristics of clusters containing one and up to five CaS units.

### 2.3. Treatments

Cells were seeded in multiple well plates and incubated for about 24 h prior to the dispersion treatment. The culture medium was removed, and the desired treatment of dispersion mixed with cell culture media was added. The maximum nanocluster concentration employed had a total calcium content of about 5 × 10^−9^ moles per 200 microliters of media for a maximum concentration of 3 × 10^−5^ M. Cell culture media was used as a control. At least three replicates per treatment combination were performed for both the CCD1090Sk (ATCC^®^ CRL2106™) and Hs 895.T (ATCC^®^ CRL7637™) cell lines. Imagining measurements included DNA label (DAPI and Hoechst/PI) and immunostaining (vinculin, actin, mitochondria, and cytochrome c). The cells were placed in an incubator at 37 °C and 5% CO_2_ for further studies. Melanoma cell cultures were also treated with dispersions of calcium acetate or zinc acetate. These dispersions contained the same amounts of acetate and cation as the dispersions of CaS nanostructures employed for the experiments.

### 2.4. Cell Viability Measurements

Human skin melanoma adherent cell cultures were prepared from the ATCC Hs 895.T CLR-7637 line. Normal human skin fibroblasts were prepared from the ATCC CCD 1090 sk CRL-2106 cell line. Cell cultures samples were prepared in 96-well microtiter plate (Costar, Arlington, VA, USA) and incubated for 24 h at 37 °C and 5% CO_2_. After this initial incubation, cells were exposed to various concentrations of CaS dispersions, which were diluted in the cell culture medium, in various concentrations that are labelled A to D. The media used for melanoma and benign skin cells were different. Phenol red was used as the pH indicator in both media. The orange color of the indicator, due to a slightly acidic media, appeared immediately after the melanoma cells are mixed with the media. This was not observed with the benign cells and the corresponding media; they maintained the reddish color typical of basic pH values until they reached maximum confluence. The cell cultures were stained with Hoechst/PI solution after 24 h and 48 of incubation, imaged in a digital fluorescence and confocal microscope with a 4× magnification, and analyzed with the microscope’s Cell Reporter Xpress Pico Image System (Molecular Devices, San Jose, CA, USA) for the cell viability measurements. The number of cells was determined from counting the nuclei stained by the Hoechst/PI solution. The number of cells alive was determined from the difference between the total number of cells and number of dead cells. Quantitative analyses were obtained with the Graph Pad package. The reported viability references the number of live cells in the sample to the corresponding number in the control experiment. Error bars represent the standard deviation from the average obtained from analysis of three to five measurements at the indicated dispersion concentration.

### 2.5. Vinculin and Actin Immunoassay

Cells were seeded in multiple-well plates and incubated for about 24 h prior to the dispersion treatment. Cells were immunostained with the Actin Cytoskeleton and Focal Adhesion Staining kit: FAK100 (Millipore Sigma, Atlanta, GA, USA) for the actin expression measurements. The cells were incubated overnight with a modified protocol that included TRITC-conjugated Phalloidin (Part No. 90228) diluted in 1% BSA; Ipswich, MA, USA 0.1% Tween^®^-20,Sigma-Aldrich, St. Louis, MO, USA, and Invitrogen™ PBS—Phosphate-Buffered Saline (10×) pH 7.4, RNase-free (PBS 1×) from Fisher Scientific, Pittsburgh, PA, USA. For the vinculin expression measurements, we used the Actin Cytoskeleton and Focal Adhesion Staining kit (FAK100) in combination with the Vinculin Monoclonal Antibody, purified clone 7F9 (Part No. 90227) and Fluorescein (FITC) AffiniPure Goat Anti-Mouse IgG (H+L) 2 mg—115-095-003 (Jackson Immuno Research, West Grove, PA, USA) with the following modifications: the antibodies were diluted in 1% BSA, 0.1% Tween^®^-20, and PBS 1×, and the primary antibodies were incubated overnight at 4 °C instead of 4 h at room temperature. An optimum working concentration of 1:250 was determined based on personal criteria of adequate cell image and an exposure time of 400 milliseconds.

### 2.6. Intrinsic Apoptosis Assay

The protocol included with the commercial ApoTrack™ Cytochrome c Apoptosis ICC Antibody—ab110417(from abcam, Fremont, CA, USA) was used without significant modifications. For immunostaining, cells were incubated overnight at 4 °C with primary antibodies (Anti-Cytochrome c monoclonal antibody (clone 37BA11) and Anti-Complex V alpha monoclonal antibody (clone 15H4C4)) for the cytochrome C and mitochondria, respectively. Both primary antibodies were diluted in 10% goat serum prior to incubation. Following primary antibody incubation, cells were incubated for 1 h at room temperature with two fluorophore-conjugated secondary antibodies. These two antibodies included Goat anti-mouse IgG2a—FITC secondary antibody and Goat anti-mouse IgG2b—TXRD secondary antibody for the cytochrome C and mitochondria imaging measurements, respectively. Rinses with 1% goat serum were performed as the manufacturers recommended. To visualize nuclei, DAPI staining was added during the final wash for a duration of 10 min. Finally, an antifade mounting medium was applied, and the prepared samples were observed using a fluorescence microscope.

### 2.7. Caspase Fluorometric Assay

The Multiplex Activity Assay Kit (Fluorometric) ab219915 was used for the determination of Caspase 3, Caspase 8, and Caspase 9 expression. Cells were seeded one day before the study to ensure proper adherence. After reaching approximately 60% confluence, the cells underwent treatment with the dispersion to induce caspase activation over a 24 h period following the manufacturer’s recommendation. The relative amount of the caspases estimated from the fluorescence intensity was determined using a spectrofluorometer (Jasco FP-8500 (ST); Easton, MD, USA). The caspase-specific excitation/emission wavelengths employed included 535/620 nm for Caspase 3, 490/525 nm for Caspase 8, and 370/450 nm for Caspase 9.

### 2.8. Cell Imaging and Processing

Cell imaging was performed with the ImageXpress Pico Automated Plate/Slide/Dish Imager (Molecular Devices, San Jose, CA, USA), which is a fluorescence and digital confocal microscope. The microscope has specialized filter cubes for FITC, TRITC, and DAPI measurements that can be employed at 40× and 63× magnifications. The CellReporterXpress image acquisition and analysis software program included with the microscope was used to quantify the amount of DNA, F-Actin, and Vinculin. The excitation wavelength of 490 nm and an emission wavelength of 525 nm for the secondary antibody (FITC) were utilized for the vinculin and cytochrome C imaging measurements. The excitation wavelength of 535 nm and an emission wavelength of 617 nm for the TRITC-conjugated Phalloidin were utilized for the actin and mitochondria imaging measurements. Finally, the excitation wavelength of 387 nm and emission wavelength of 470 nm were utilized for DAPI for nucleus imaging measurements.

## 3. Results

### 3.1. Benign Cell Cytotoxicity

**Figure 1:**
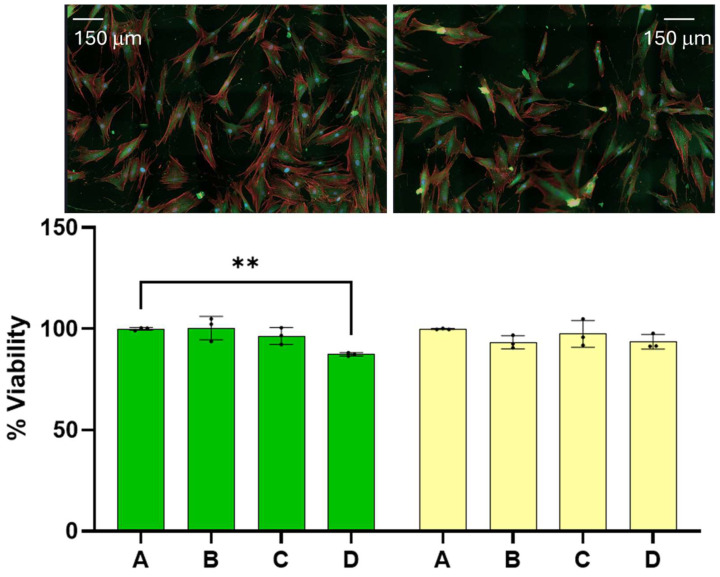
The effect of the dispersion on benign cells is reported here. Fibroblasts treated with the dispersion did not develop significant differences from the corresponding control. The 24 and 48 h viability of benign cells as a function of dispersion percentage (%) are indicated by the green and yellow bars, respectively. The (**) represents a *p* value < 0.01.

At the onset, we wish to report that the amounts of dispersion employed for the experiments reported here have little effect on the viability of benign cells. The 24 and 48 h viability of benign cells as a function of dispersion percentage in the media are summarized on the left- and righthand side of Figure 1, respectively. Reported viability represents the percentage of cells alive referenced to the average number of cells found alive in the control experiment. Dispersion percentage was varied by simply adding different dispersion volumes (of the order of microliters) to the media employed to feed the cells. Dispersion percentages employed are A = 0%, B = 1.0%, C = 2.0, and D = 2.5%. The 24 and 48 h viabilities were found to remain nearly constant within experimental uncertainty in the range of dispersion concentrations employed. The 48-h viability of benign cells was in the range of (100.0 ± 0.2) for the control and (93 ± 2) when the dispersion B was employed. The viability of the benign cells at the highest dose employed was found to be (94 ± 2)%. These ranges of values are significantly close to each other, and an ANOVA test of the measurements did not allow us to distinguish among the four measurements, with a *p* < 0.05. The viabilities of dispersions B and D were distinguished from the control at a *p*-value < 0.1, which is typically considered higher than the standard *p*-values employed in this type of measurement. We did not observe any changes in the physical characteristics of the benign cells. These results lead us to conclude that the range of dispersion concentrations employed has little effect on benign cell viability at 24 and 48 h post dose with the dispersion concentrations employed here. These ranges of viabilities are lower than those reported for other chemotherapeutic agents under FDA evaluation or commercially available medications and drugs [19,20].

### 3.2. Mesenchymal and Epithelial Melanoma

Melanoma cells with epithelial phenotype frequently exhibit a distinctive arrangement of G-actin localized in the cortex below the plasma membrane. G-actin is involved in various cellular processes, including muscle contraction, cell motility, and cellular shape. In contrast, the mesenchymal phenotype is characterized by actin filaments or F-actin, which are formed from globular actin polymerization. Mesenchymal cells have a dispersed or non-homogeneous distribution of actin throughout the cytoplasm, forming stress fibers and other filamentous structures. This organization contrasts with the G-actin seen in epithelial cells discussed above. The arrangement of F-actin in the melanoma cells with mesenchymal phenotype facilitates cell migration and plays an important role in the characteristic elongated cell shape.

**Figure 2:**
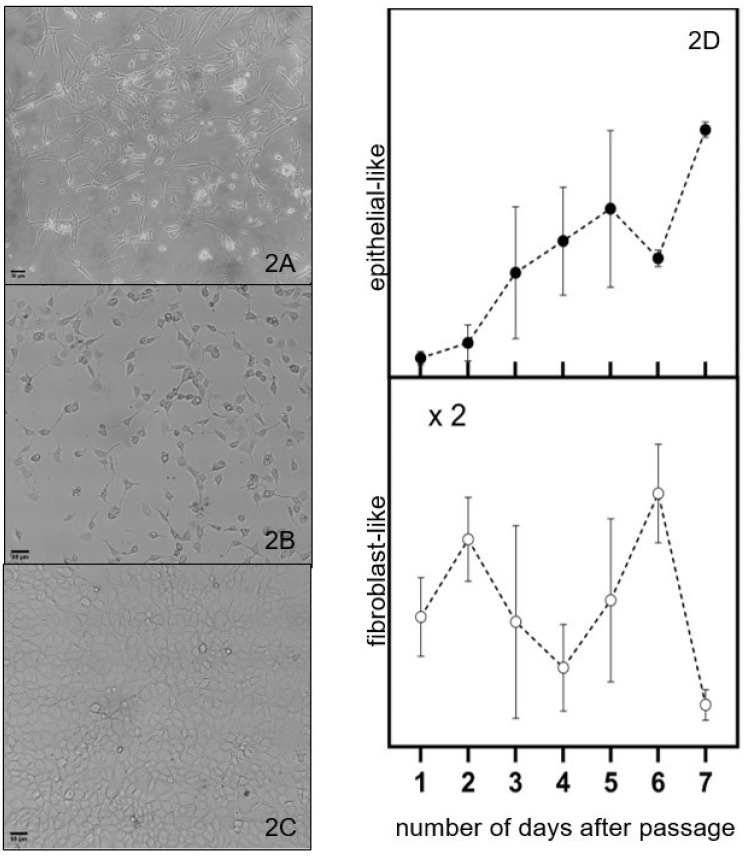
Optical microscopy images of melanoma cells with (**A**) predominantly fibroblast phenotype, (**B**) mixture of cells with fibroblast-like and cuboidal-like morphologies, and (**C**) cells of predominantly epithelial phenotype at maximum confluence. The fraction of cells with fibroblast-like and cubic-like morphologies as a function of days after the passage is represented by the open and closed circles in (**D**), respectively. The cut lines are to guide the eye through the discussion and do not represent a model fit to the data. Scale bar: 50 μm (**A**–**C**).

The Hs 895.T (ATCC^®^ CRL7637™) line was found to be predominantly melanomas with the mesenchymal-like phenotype as received and used after a handful of passages, illustrated on Figure 2A. These cells are wide in the middle and become thinner towards the two ends. A mixture of cells with shapes similar yet not identical to the ones reported for epithelial- or mesenchymal-like phenotypes cells was observed after about forty (40) passages from the initial seed of the received cell line, as illustrated on Figure 2B. We refer to these cells as having a cuboidal-like or fibroblast-like morphology. This trend can also be observed in new cell cultures that are grown after several freeze–thaw cycles. Cell cultures close to maximum confluence have a cuboidal-like morphology, as illustrated in Figure 2C.

The fraction of cells with fibroblast-like and cuboidal-like morphologies as a function of days after a passage is represented by the open and closed circles in Figure 2D, respectively. The general trend observed is the increase in the fraction of cuboidal-like melanoma cells at the expense of a decrease in the fraction of fibroblast-like cells with the number of days after a passage. This trend suggests a shift towards a more proliferative state, potentially impacting tumor growth and response to therapies. This transition can be influenced by various factors, including cell environment, signaling pathways, and genetic alterations [21]. Prospective treatments must be effective to reduce proliferation and growth of mesenchymal and epithelial melanoma cells.

### 3.3. Melanoma Cell Cytotoxicity

Melanoma cells with predominantly epithelial-like phenotype were observed after conducting 15 and 20 passages of the initial cells received from ATCC. These melanomas reproduced quickly to form clusters containing about 3 to about 15 cells.

**Figure 3:**
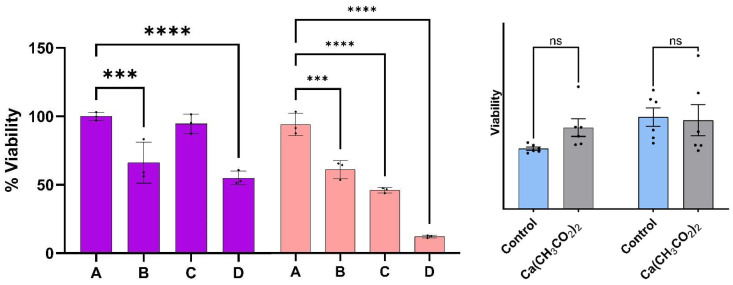
The viability of melanoma cells with epithelial-like phenotype as a function of the dispersion percentage (%). Viabilities determined at 24 and 48 h post treatment are summarized on the left- and right-hand side respectively. Error bars represent standard error. The symbols *** and **** represent a difference in *p*-values of 0.001, and 0.0001, respectively, among the treatment concentrations employed. Dispersions of Ca(CH_3_CO_2_)_2_ in DMSO with the same concentrations as the one employed to prepare the CaS dispersions employed for the experiments described here did not result in viability differences that were statistically significant (ns) from the corresponding control at 24 or 48 h post dose.

Prior to discussion of the viability measurements described below, the viability of melanoma cells with the epithelial-like phenotype as a function of the treatment percentage determined at 24 and 48 h are summarized on the left- and righthand side of Figure 3, respectively. The treatment percentages fed to the cell cultures ranged from A = 0% (control) to D = 3%. Viabilities were obtained from fluorescence microscopy measurements of stained nuclei (Hoechst dye) at 24 and 48 h post treatment. Reported values represent the average of three to five biological replicates at the same times and dose. Viabilities of the malignant cell culture at the indicated times without treatment (A) are used as control experiments. The viability of malignant cells reached values as low as (55 ± 5)% and (13 ± 1)% at 24 and 48 h post treatment, respectively, when the dispersion of concentration D was used; these values are statistically different from those observed in the melanoma control at the same times. A linear regression analysis of the average viability as a function of dispersion % resulted in a straight line with an intercept of 98.4 and a slope of −27.6 with a R^2^ of 0.98. Using this relationship, we estimated that the 48 h IC 50 has about a 1.75% volume of dispersion per culture media.

### 3.4. Cytochrome C Expression

**Figure 4:**
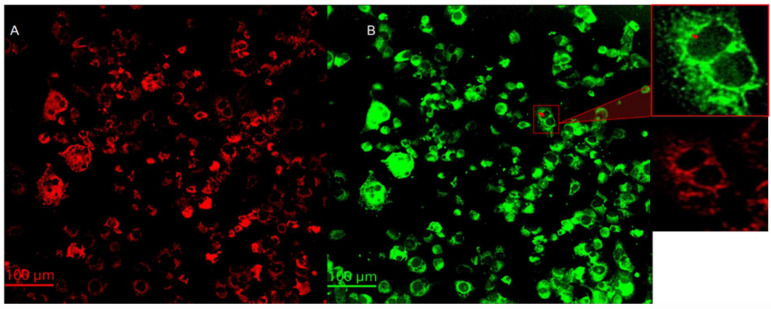
The left- (**A**) and right (**B**) hand side summarize the mitochondria and cytochrome c of melanoma cells with the epithelial-like phenotype at 24 h post treatment. The objective magnification is 20×. The insert at the upper right-hand corner represents a 200 × 200 m^2^ region, magnified by a factor of about 4, of the indicated 24 h measurement. The red arrow indicates cytochrome c release from mitochondria.

Cytochrome C is a protein localized inside the mitochondria. One of the most important functions of cytochrome C is to facilitate the transfer of electrons between complex III and complex IV in the respiratory chain [22]. Cytochrome C is also known to play important roles in programmed cell death, particularly in intrinsic apoptosis. After mitochondria is permeabilized by Bad/Bax protein complexes, cytochrome C is released from the intermembrane space of the mitochondria to the cytosol [23]. Cytochrome C then activates caspase enzymes, initiating a cascade of proteolytic events that ultimately result in cell death [24] Images of mitochondria (red) and cytochrome C (green) are summarized on the left- (A) and righthand (B) sides of Figure 4, respectively. Anti-ATP synthase subunit alpha and anti-cytochrome C antibodies were used to label the mitochondria and cytochrome C, respectively. There is a significant co-localization of cytochrome c in the mitochondria. However, careful evaluation of the images reveal that cytochrome C is also present in regions outside the mitochondria and the nuclei, as shown in the 200 × 200 μm^2^ insert on the righthand side of Figure 4. The red arrow indicates the cytochrome C released from the mitochondria 24 h post treatment. The translocation of cytochrome c from the mitochondria to the cytoplasm and nuclei is consistent with the activation of intrinsic apoptotic mechanisms [25].

### 3.5. Caspase Expression Measurements

**Figure 5:**
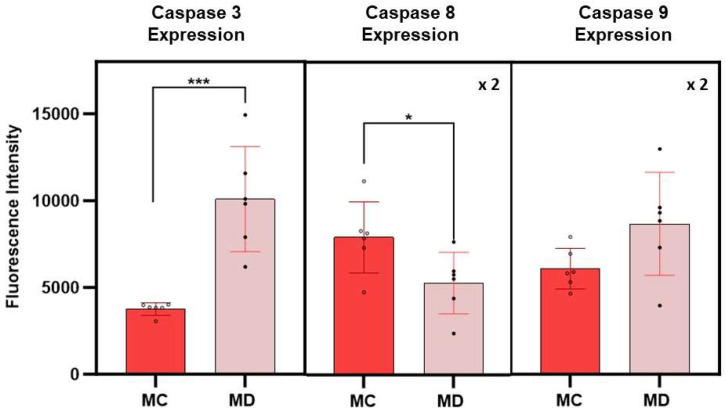
Average fluorescence intensities corresponding to caspases 3, 8, and 9 expressions in melanoma cells with epithelial-like phenotype at 24 h post treatment with the dispersion D. MC and MD represent melanoma control and melanoma dispersion, respectively. Error bars represent the standard error. The fluorescence intensity of caspases 8 and 9 was multiplied by a factor of 2 for purposes of using the same scale for all measurements. The symbols * and *** indicate a significant difference in *p*-values between MC and MD (the number of * characters correspond to the decimal places in reported values.

The expression of caspases 3, 8, and 9 in melanoma cells with the epithelial-like phenotype is summarized in Figure 5. Caspase expressions were obtained from fluorescence spectroscopy measurements of cell culture supernatants following 24 h post treatment with the D dispersion. Results on the fluorescence measurements corresponding to caspase assays from melanoma cells with the epithelial phenotype without treatments were used as control experiments. The average fluorescence intensity of caspase 3 was found to be more than 2.5 times higher in treated melanoma cells than in the corresponding control cell (*p* > 0.005). The data corresponding to the fluorescence intensity of caspases 8 and 9 have a significant scatter that results in average values with higher standard errors, limiting the interpretation of the results; however, the average value of caspase 9 was higher than the corresponding control. Early death events in the cell changed the sensitivity of the assays, rendering the caspases 8 and 9 signal intensity of less value to differentiate among extrinsic and intrinsic programmed death cell process. If this is indeed the case, further experiments at shorter times will be of interest to elucidate the role of these caspases in the apoptotic events related to the effect of these engineered nanoclusters in melanoma cells.

### 3.6. Vinculin and Actin Expression

**Figure 6:**
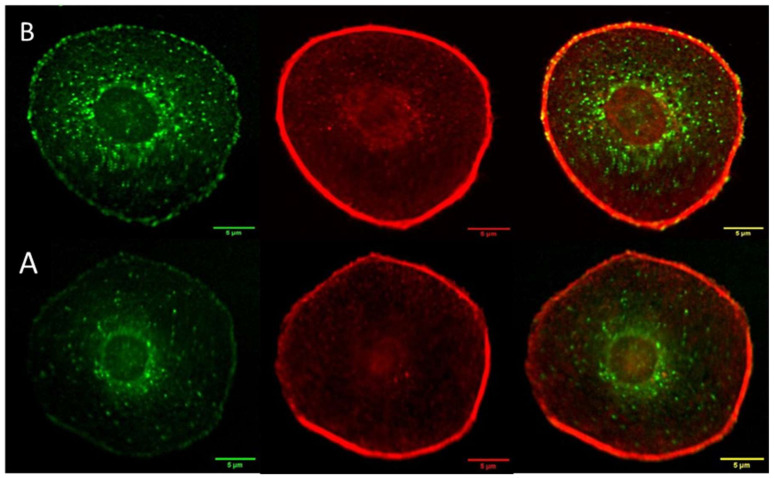
Digital confocal fluorescence microscopy at 60× magnification of vinculin (green), actin cytoskeleton (red) and focal adhesion (composite of vinculin and actin expression) in Hs 895.T (ATCC^®^ CRL7637™) cells in (**A**) control and (**B**) 24 h post treatment. Focal adhesions were detected using an anti-vinculin monoclonal antibody and an FITC-conjugated secondary antibody; actin was detected using TRITC conjugated Phalloidin. The delocalization of vinculin in the cytoplasm is taken as evidence of FAK regulation in post-treatment malignant cells. Focal adhesion points localized on the interphase with the ECM increase, as evidenced by the bright spots on the cell membrane. Scale bar is 5 µm.

The vinculin and actin expression of melanoma cells with the epithelial-like phenotype 24 h post treatment is represented by the green and red areas in the left and center panel of Figure 6. Localized clusters of vinculin near the nuclei are easily identified in both treated and control melanoma cells exhibiting the epithelial phenotype. There is little co-localization of the vinculin- and actin-expressed regions close to the nuclei. This is not surprising since vinculin does not bind directly to globular actin, which is the dominant form in epithelial cells [26].This observation reflects the importance of vinculin participation in cellular processes that do not have a direct association with actin. These processes may include modulation of integrin signaling and nuclear signaling, which can influence gene expression, cell cycle regulation, and cell proliferation and survival [27]. No statistically significant difference was found between the average number of vinculin clusters in either the neighborhood of the nuclei of cell cytoplasm in treated melanoma and control cells.

Co-localization of vinculin and actin was observed at the cell membrane and extracellular matrix (ECM) which is usually associated with focal adhesion points (FAP). FAP are complexes that connect the cell’s cytoskeleton to the extracellular matrix, providing stable anchors. Increasing focal adhesion points generally favors cell adhesion over cell mobility [28,29].

**Figure 7:**
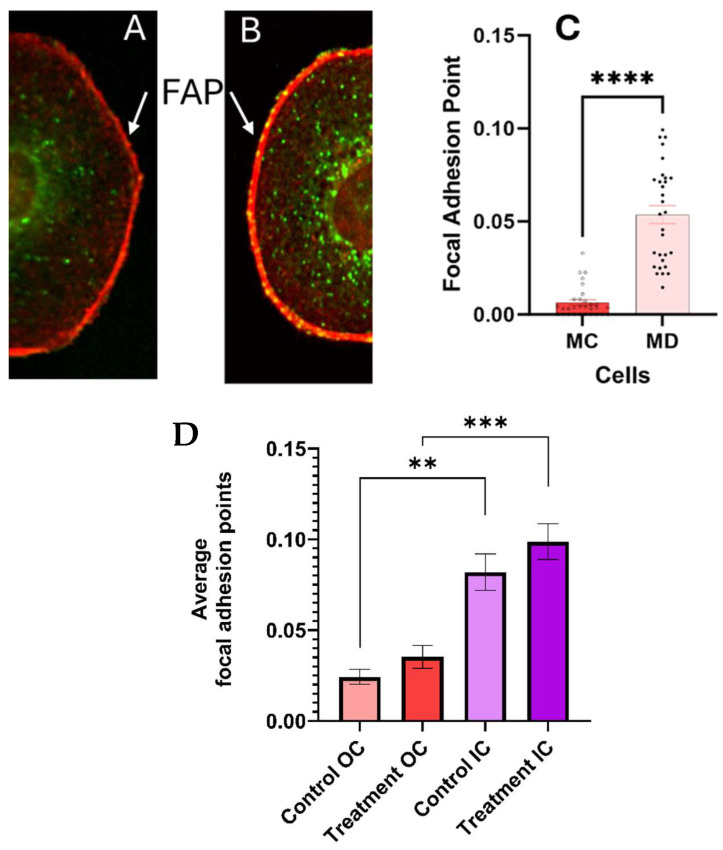
The dispersion increases the number of bright focal adhesion points (FAP) at the cell membrane–ECM interphase in melanoma cells 24 h post treatment, as summarized in (**A**–**C**) (*p* < 0.0001). The FAP close to the nuclei (IC) and at a distance of a 1.75 times the nuclear ratio (OC) are statistically unchanged in the control and treated melanoma cells, as summarized in (**D**). The error bars in the graphs represent the standard error of measurements within a 95% confidence limit. The number of asterisks (*) corresponds to the level of significance and number of decimal places in the *p*-value.

**Quantitative measurements of the number of FAP** in the cell membrane with ECM are summarized in Figure 7. The standard error measurements were used as the uncertainty of the measurements. Statistically significant differences were found (*p* < 0.0001) in the number of FAP in the cell membrane–substrate support interphase between the melanoma cells with epithelial phenotypes 24 h post treatment and the corresponding control. We conclude that treatment with the CaS dispersion increases the number of FAP and anchoring to the substrate.

### 3.7. Cell Cytotoxicity in Melanoma Cells with Mesenchymal-like Phenotype

**Figure 8:**
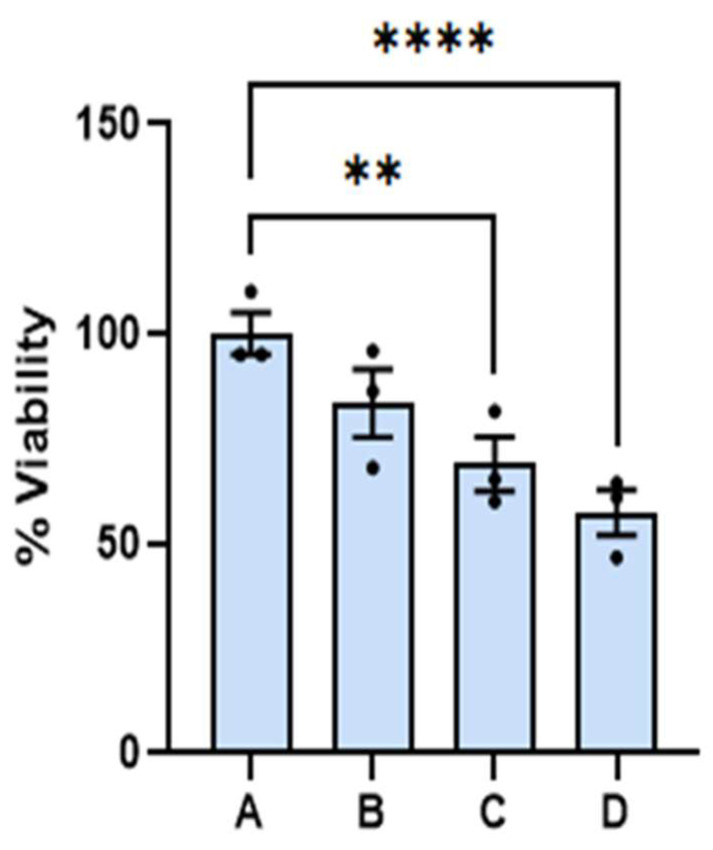
The 24 h viability of melanoma cells with mesenchymal phenotypes as a function of the percentage (%) of dispersion used. Error bars represent the standard error with confidence level of 95%. The (**) and (****) represent *p* values < 0.01 and <0.0001, respectively.

The results of the cell viability measurements in melanoma cells with the mesenchymal phenotype are summarized in Figure 8. Cell viability decreased with treatment concentration until it reached about (57 ± 5)% with dispersion D at 24 h post treatment. A linear regression analysis of the average viability as a function of dispersion % resulted in a straight line with an intercept of 99.0 and a slope of −15.0 with a R^2^ of 0.978. Using this data, we estimated that the 24 h IC 50 has about a 3.3% volume of dispersion per culture media.

**Figure 9:**
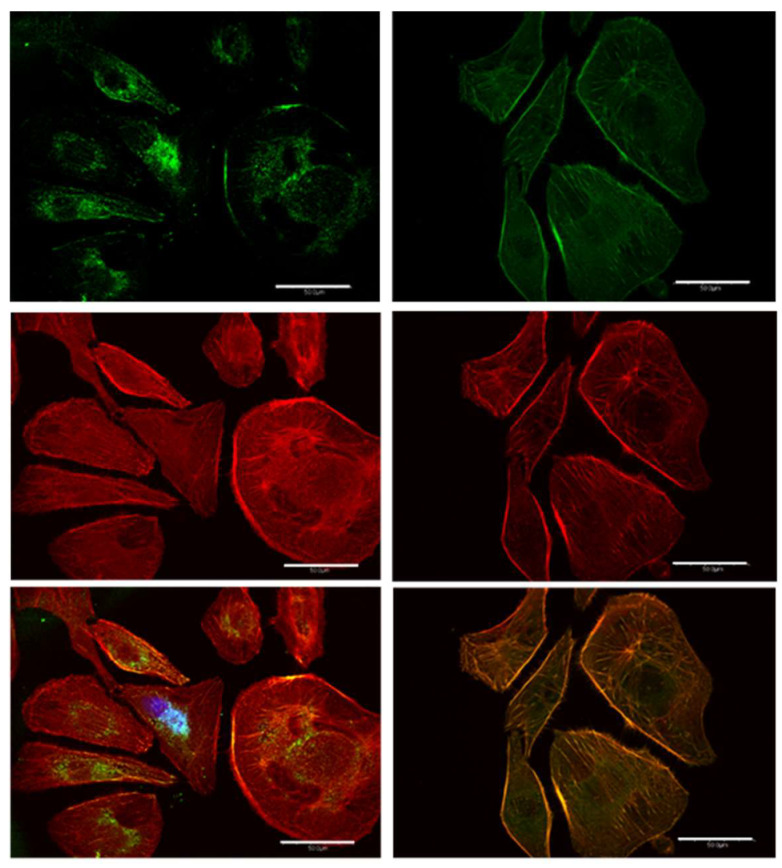
The dispersion facilitates the regulation of relevant proteins associated with FAP. Digital confocal fluorescence microscopy at 60× magnification of vinculin (green) and actin (red) expression in Hs 895.T (ATCC^®^ CRL7637™) melanoma with a predominantly mesenchymal phenotype. The images corresponding to the control and 24 h post treatment are indicated at the left- and righthand sides of the figure, respectively. The delocalization of vinculin in the cytoplasm is taken as evidence of FAK regulation in post-treatment malignant cells. The indicated bar length corresponds to 50 μm.

**Vinculin (green) and actin (red) expression in melanoma cells** with the mesenchymal-like phenotype are summarized in Figure 9. Vinculin in the nuclear neighborhood, well-defined actin fibers, and the elongated shape are characteristics of melanoma cells with the mesenchymal phenotype. Vinculin expression in the control cells is highly localized close to the nuclei, with significant co-localization with actin, particularly close to the nuclei. Images of vinculin and actin expression in these mesenchymal melanomas 24 h post treatment results in delocalization of vinculin in the cytoplasmic fluid and increased colocalization of vinculin and actin, as evidenced by the larger orange area in the composite image. The emergence of well-defined co-localization of vinculin and actin in the extracellular matrix (ECM) of treated mesenchymal cells is a significant difference compared to the trend observed in the control experiments.

## 4. Discussion

**Figure 10:**
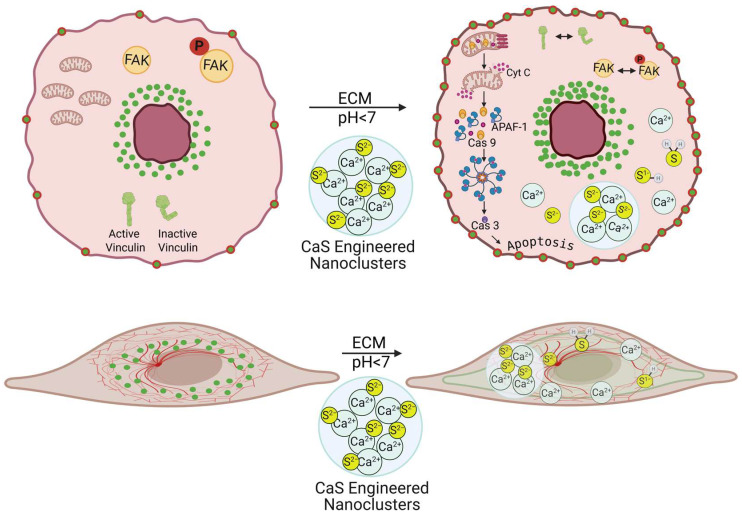
Scheme to guide the eye through the discussion. The viability of melanoma cells with predominantly the epithelial or mesenchymal phenotype is reduced by the activation of CaS nanoclusters. The resulting sulfide distribution is proposed to enter the malignant cells, where the regulation of key proteins promotes intrinsic apoptosis.

At the onset, we refer the reader to the scheme in Figure 10 to guide the eye through the discussion. Many cancer treatments have an adverse impact on healthy cells, which causes undesirable secondary effects that may be as severe as organ damage. We found that the CaS nanoclusters have no effect on the viability of benign cells. The same concentrations of CaS nanocluster dispersion result in reduced viability of melanoma cells. The viability of melanomas with the epithelial and mesenchymal phenotypes was reduced to about (55 ± 5) and (57 ± 5)%, respectively, within 24 h post treatment. The viability of melanomas with the epithelial phenotype was reduced to (13 ± 1)% at 48 h post treatment. The selectivity of the CaS to affect the viability of melanomas with little effect on the viability of benign fibroblasts highlights the potential of this nanotechnology with little modification as an active ingredient to treat melanomas [30]. Along these lines, the findings reported here are consistent with our previous research with lung and breast malignancies [13,14,17], where the viability of cancer cells was found to selectively decrease, with little or no significant effect on the viability of benign cells. We propose that the acidic extracellular pH of cancer cells facilitates the release of Ca^2+^ and sulfides according to the following:

CaS_(s)_ + 2H^+^_(aq)_ → Ca^2+^_(aq)_ + H_2_S_(aq)_ (1)

The formation of H_2_S from CaS_(s)_ in acidic but not basic environments has been documented by us [13,14,16]. The H_2_S is predicted to form a distribution of sulfides that include H_2_S_(gas)_, H_2_S_(aq)_, HS^−^_(aq)_, and S^2−^_(aq)_. Recent work from our group determined an increase in Ca^2+^_(aq)_ concentration in the supernatant of non-small-cell lung carcinoma (NSCLC) cultures treated with CaS dispersions similar to the ones used here [17]. The sulfide distribution and the Ca^2+^ ions can also participate in events that result in cell death [18].

Calcium ions are part of the cocktail of chemicals formed upon dissociation of the calcium sulfide nanoclusters. Calcium ions are also necessary components of membrane polarization, which is associated with cytochrome c release [22,23,24,25]. It is appropriate to highlight the results related to the effect of calcium acetate in the viability of melanoma cells. The results of the viability measurements of cell cultures treated with calcium acetate in DMSO summarized in Figure 3 suggest that the concentrations of the calcium precursor employed for the nanocluster synthesis are too small to facilitate cell death. By contrast, the viability of melanoma cells treated with the calcium sulfide nanostructures was reduced to (13 ± 1)% at 48 h post dose. We conclude that the calcium ions released by the nanostructures in the experiments reported here are not large enough to initiate intrinsic apoptosis.

Damage to the cell mitochondria results in the release of cytochrome c. Evidence for the release of cytochrome c to the cell cytoplasm and translocation to the nuclei is summarized in Figure 4. Initiator caspase 9 is activated during the intrinsic pathway by released cytochrome c. Indeed, the average expression of caspase 9 in treated melanomas was found to be higher than the corresponding expression in the control melanoma cultures. In general, the executor caspase 3 is activated by the apoptosome formed by the three-way interactions among cytochrome c and caspase 9 and APAF-1 [22,23,24,25]. The expression of caspase 3 is statistically higher by a factor of three in treated melanomas than in the corresponding control. Thus, the experimental measurements are consistent with the intrinsic apoptotic pathway to cell death.

Proto-oncogene tyrosine-protein kinase (SRC) and focal adhesion kinase (FAK) regulate the intrinsic apoptotic pathway by modulating key proteins such as the BCL-2 family members, including BAX and BAK, and p53, which control the permeabilization of the mitochondrial membrane that facilitates the release of cytochrome c [31]. SRC and FAK also regulate key intracellular signaling pathways that support melanoma cell survival, proliferation and migration [32]. In cancer cells, the FAK and SRC are not properly regulated, which results from improper phosphorylation/dephosphorylation of tyrosine residues [32]. Tyrosine dephosphorylation is generally accepted to be initiated by sulfide-rich cysteine residues [33] Along this line, the engineered nanostructures can provide sulfides that may substitute this cysteine function and provide an alternative mechanism to tyrosine dephosphorylation. These sulfides may include the distribution of H_2_S, both in solution and gaseous, and HS^−^ and S^2−^ ions as well as partially unreacted clusters that may have entered the cell after surviving the acidic extracellular environment of melanoma. These species may facilitate dephosphorylation and appropriate FAK/SRC regulation. In addition to intrinsic apoptosis, evidence for regulation of these proteins initiated by the treatment includes the increase in the number of focal adhesion points (FAP) in the cell membrane of melanoma cells with epithelial phenotypes. Melanoma cells with the epithelial phenotype exhibit focal-adhesion-bound vinculin, which is not colocalized with actin, in a gradient from the cell membrane to the cytoplasm and immediate outer regions of the nuclei [34,35]. The nanocluster dispersion has no effect on these focal adhesions in melanomas with the epithelial-like phenotype. In cells with the mesenchymal-like phenotype, on the other hand, the nanocluster treatment delocalizes vinculin bound to actin in FAP in the cytoplasm. Future work using Western blots may strengthen our understanding of the role of sulfides in protein dephosphorylation.

This difference in the responses of the two distinct melanoma phenotypes to the dispersion is intriguing. **Vinculin localization, interactions, and functions** can differ significantly due to the different cellular environments [36,37]. These differences can result in conformational changes in vinculin structure. In epithelial melanomas, vinculin may be in a closed conformation that facilitates cell-to-cell junctions and tissue cohesion [34]. Relevant interactions of vinculin with cadherins, a and b catenins, p120-catenin, and actin in the epithelial cells facilitate this function. In mesenchymal cells, on the other hand, vinculin has an open and dynamic conformation that facilitates interaction with proteins related to cell migration, focal adhesion points (FAP), and ECM interactions [38]. These proteins include integrins, paxillin, talin, and actin. Pinpointing the nature of the nanostructure or sulfide interactions that result in the observed vinculin response is beyond the scope of the present work and deserves further research [38]. In passing, we note that FAK and SRC also play an important role in the mesenchymal-to-epithelial transition. Cell cultures with the epithelial- and mesenchymal-like phenotypes resulted in similar viabilities at 24 h post dose. Thus, the dispersion appears to have a more important role in promoting cell death than affecting the mesenchymal-to-epithelial transition. This is an important observation. The melanoma MET-EMT among phenotypes facilitates survival against chemotherapies and the individual’s own immune system.

The discussion in the previous paragraphs focused on apoptosis to account for the decrease in viability of the two distinct melanoma phenotypes with dispersion treatment. Some of our observations may also occur in other cell death processes, like necrosis or ferroptosis. Vinculin delocalization, for instance, may occur in other processes where changes in the cell skeleton are associated with cell death. It is generally accepted that necrosis and ferroptosis are caspase-independent processes, in sharp contrast with our observations. Nanosulfides may also bring new cell death mechanisms that may contribute to the reduced viability of melanomas.

The principal aim of any study to treat cancer is to develop active ingredients that reduce cancer cell survival, with little minimal secondary effects. Recent progress in the development of redox-sensitive nanocarriers in the size range of 50 to about 200 nm target the high concentration of glutathione present in melanoma cells [39,40]. Tumor heterogeneity is a major limitation of redox-sensitive nanocarriers. Since benign cells also have glutathione, there is a risk of developing secondary effects in non-malignant tissues. Furthermore, the design of carriers with precise redox responsiveness and good pharmacokinetics can involve multiple synthetic steps, which may result in materials that are difficult to scale up for clinical use. Integrin-targeting systems have shown great promise for melanoma-targeted drug delivery, but they also face several key limitations, including saturation of the integrins and shielding of the integrins in the tumor itself, variations in integrin expression among patients, and the activation of the immune system, which may result in fast elimination by the body [41,42]. Since healthy cells also have integrins, this technology may also result in secondary effects on benign tissue. In contrast, the nanotechnology discussed here takes advantage of a simple chemical process and does not affect the viability of benign cells studied.

## 5. Conclusions

In summary, we studied the effect of nanoclusters on the viability of melanoma cells with epithelial- and mesenchymal-like phenotypes. We found a 24 h viability slightly over 50% in melanoma cells of each phenotype. An increase in the number of actin fibers was observed 24 h post treatment of melanoma cells with the mesenchymal-like phenotype (Figure 8), while a trend in the opposite direction was seen for the corresponding mesenchymal control. In contrast, melanomas with the epithelial-like phenotype did not exhibit significant changes in the globular actin distribution compared to their control. Vinculin delocalization was evident in melanoma cells with both phenotypes 24 h post treatment, yet they exhibited different interactions with actin. Mesenchymal melanoma cells displayed increased vinculin co-localization with actin. The vinculin delocalization in melanomas with the epithelial cell phenotype led to a significant increase in focal adhesion points at the interphase of the cell membrane and ECM (Figure 6).

## Data Availability

The data presented in this study are available in this article.
